# The lived experiences of mothers who have children with congenital abnormalities in the Gert Sibande district

**DOI:** 10.4102/curationis.v45i1.2250

**Published:** 2022-07-26

**Authors:** Thandeka B. Mazibuko, Tendani Ramukumba, Nomusa Ngwenya

**Affiliations:** 1 Adelaide Tambo School of Nursing Science, Faculty of Science, Tshwane University of Technology, Pretoria, South Africa

**Keywords:** congenital abnormalities, lived experiences, children, mothers, Gert Sibande

## Abstract

**Background:**

Mothers are regarded as primary care givers. The experience of having a child with congenital abnormality may have an impact on their psychological well-being. It was observed that the psychological well-being of mothers is often unattended by health professionals, including nurses and their families. Mothers make adjustments in their daily lives to ensure the child’s activities of daily living are attended. Therefore, this raises the need for the experience of mothers who have children with congenital abnormalities in the Gert Sibande district to be explored. Gert Sibande was chosen because it has been observed that several mothers who visit the clinic and outpatient department have children with congenital abnormalities.

**Objectives:**

The purpose of this study was to explore and describe the lived experiences of mothers who have children with congenital abnormalities.

**Method:**

A phenomenological study was conducted. Purposive sampling of 12 participants was done. The study used adjusted ecological model of health for guidance. Data gathering was done by self-report using unstructured face-to-face interviews until data saturation was reached.

**Results:**

Five themes emerged from the study, which include ‘being hurt emotionally’, ‘sense of guilt’, ‘acceptance’, ‘support from family’ and ‘community reaction to the congenital abnormality’. The findings show that mothers were affected by having children with congenital abnormalities.

**Conclusion:**

The study revealed that mothers of children with congenital abnormalities experienced devastation, denial, guilt and lack of acceptance of their child’s condition. The study indicates that mothers require support from health care professionals, family and the community.

## Introduction

There are several children with congenital abnormalities seen at clinics and hospital outpatient departments in the Gert Sibande district. Mothers in rural areas are more likely to be malnourished before and during pregnancy and are at greater risk of exposure to environmental teratogenic factors, such as alcohol and maternal infections (Labaran 2020:7). Meanwhile, despite the availability and accessibility of health care services mothers may still delay attending antenatal clinics. In the Gert Sibande district, mothers are exposed to traditional remedies, which are not scientifically known to contain teratogens. This may lead to congenital abnormalities, such as Down syndrome, cerebral palsy and hydrocephalus, which were not detected before delivery. The study explored the lived experiences of mothers who have children with congenital abnormalities at a hospital in Gert Sibande District.

McFerran (2014:154) defines congenital abnormality as a condition that is recognised at birth or that is believed to have been present since birth. Malherbe et al. (2016:149) state that congenital disorders are not recognised as a health issue in South Africa. Therefore, the contribution of congenital abnormalities to the disease burden is underestimated. During pregnancy, mothers expect a healthy foetus, anticipate giving birth to a healthy baby and carry a foetus for 9 months with the hope of bringing about great change in the family. The birth of a child is normally an experience filled with joy and happiness. However, according to Bananno, Bennett and Pitt (2013:100), some parents may have a different experience and meaning when their children are born with one or more congenital abnormalities. Finding that their children have a congenital abnormality can have an effect on parents’ physical, psychological and mental well-being.

Barr, Govender and Recken (2016:934) state that the birth of a child with a congenital abnormality resulting in physical disability has been linked with cultural opinions of the community ascribing negative life events to bad spirits or inadequate cultural practices. According to Honikman et al. (2012:2), in African countries, women are culturally held responsible for a child’s health. According to Huiracocha et al. (2017:493), mothers are the spiritual hub of the family, responsible for care of the children and household tasks.

According to Lemacks et al. (2013:3467), mothers may become devastated when they learn that their child has a congenital abnormality, which may be during pregnancy, upon delivery or during the child’s early life. In addition, these mothers experience psychological stress and disappointment when their child does not meet their hopes and expectations of a healthy child. Adequate support from the partner, family and health providers is essential during this period. This will help reduce the influence of psychological effects of congenital abnormality on the mother and increase the acceptance of the child.

Lemacks et al. (2013:3467) state that mothers often go through stages of grief after a child has been diagnosed with a congenital abnormality. These stages can be similar to those they would have experienced had they lost the child. According to Barr et al. (2016:934), mothers may experience feelings of fear, guilt, frustration, uncertainty, anger, sadness and loss.

## Problem statement

The perinatal period is regarded as a treasurable and exciting period for women. However, Honikman et al. (2012:4) state that during this time, women are vulnerable to psychological breakdown from a social, economic and gender-based perspective. It was observed that in the rural Gert Sibande district, women who have delivered children with congenital abnormalities usually lack support and could suffer from emotional breakdown and therefore can be prone to psychological breakdown. The research problem was that mothers’ experiences of having children with congenital abnormalities are not known. Therefore, the research question was, ‘what are the experiences of mothers who have children with congenital abnormalities?’

## Purpose of the study

The purpose of this study was to explore and describe the lived experiences of mothers who have children with congenital abnormalities.

## Research methods and design

### Research design

An exploratory descriptive phenomenological design was used in this study. Neubauer, Witkop and Virpio (2019:90) define phenomenology as a form of qualitative research that focuses on the study of an individual’s lived experience. The approach seeks to describe the essence of a phenomenon by exploring it from the perspective of those who have experienced it. This design was applicable to this study because the study determined and described the lived experiences of mothers who had children with congenital abnormalities in the Gert Sibande district. In this study, the mothers explained and expressed how having children with congenital abnormalities impacted their lives.

The theoretical framework of the study was ecological model of health. According to Glanz, Rimer and Viswanath (2015:465), the theory emphasises the environmental and policy context behaviour, whilst incorporating social and psychological influences. The core concept of ecological model is that behaviour has multiple levels of influences, including individual, interpersonal, organisational, community and public policies. There are multiple levels of influence on specific health behaviours: the behaviour of mothers was influenced by rejection and disappointments. Factors that influenced the health behaviour of mothers included self-blame, guilt, frustration, denial and dissatisfaction. The theory also emphasises on behaviour across the different levels. Moreover, ensuring that community centres cater for children with congenital abnormalities as well as offering support and care for the mothers.

### Study setting

This study was conducted at a regional hospital in Mpumalanga. The hospital was chosen because it is a regional and referral hospital for all other eight district hospitals in the Gert Sibande district. Therefore, all high-risk pregnancies are referred to this hospital for a higher level of care. Participants were recruited via the well-baby clinic, paediatric outpatient department. The maternity register was used to identify the potential participants.

### Study population and sampling

Mothers who were aged 18 years and older and who had children with a congenital disability aged 3 years and younger were included in the study. Purposeful sampling was used to recruit participants. The estimated number of mothers who attended clinic at the feeder was 60. Sample size was determined by saturation, which was reached after 12 participants were interviewed. The demographic information of participants is presented in Table 1. Pseudonyms were used instead of real names of participants as a form of protection of participants’ identity.

**TABLE 1:** Demographic information of participants.

Pseudonyms of participants	Age (years)	Educational level	Type of child’s congenital abnormality
1. Thabile	26	Grade 12	Short fingers (left hand)
2. Anele	20	Grade11	Down syndrome
3. Zodwa	30	Grade 12	Cerebral palsy
4. Mbali	21	Grade11	Cerebral palsy
5. Maria	40	Grade 8	Cerebral palsy
6. Thobeka	22	Grade 12	Cerebral palsy
7. Bongiwe	32	Grade 12	Cerebral palsy
8. Ntonkozo	36	Grade 10	Cerebral palsy
9. Betty	44	Grade 7	Down syndrome
10. Nomthandazo	20	Grade 12	Down syndrome
11. Thembi	27	Grade 12	Hydrocephalus
12. Thembeka	35	Grade 12	Down syndrome

### Data collection procedure

Permission was obtained from the Department for Postgraduate Studies, Faculty Committee for Postgraduate Studies and Research Ethics Committee of the Tshwane University of Technology. Permission to conduct the study was obtained from the Ermelo Hospital. Permission was also obtained from the Mpumalanga Department of Health Research Committee.

An arrangement with the hospital management was done. The researcher approached mothers who have children with congenital abnormalities attending the outpatient department. The researcher explained to potential participants what the study entails. Mothers who were willing to participate were requested to sign consent forms. Data were collected over a period of 2 months.

Data were gathered through unstructured individual interviews conducted by the researcher. Permission to use audio recordings was obtained from participants. Data were primarily gathered in Zulu as mothers felt they could express themselves better in their own language. Data were collected in a private place; a room was provided to the researcher. One central question was asked: ‘what is your experience of having a child with congenital abnormality?’ Probing and clarity seeking questions followed. Interviews lasted for 30–40 min. Data saturation was reached after 12 participants were interviewed.

### Data analysis

Data analysis is performed to reduce, organise and give meaning to data (Burns, Grove & Gray 2013:690). Data analysis was done using Tesch’s approach to identify emerging themes and subcategories. The verbatim transcriptions were analysed after saturation was reached. Verbatim transcriptions were done from audio-recordings to paper. Interviews were translated from IsiZulu to English. The researcher is first language Zulu speaking and could clearly understand what the participants were trying to express. A Zulu speaking co-coder was employed to listen and read the transcript. The researcher read the transcripts more than once to get the true meaning and wrote notes and arranged feelings expressed. Furthermore, researcher identified subcategories and later group similarly repeated codes into categories, categories were later merged to form themes. The researcher and the independent co-coder shared the findings, confirmed the themes and categories. Trustworthiness was ensured by credibility, dependability, transferability and conformability. A friendly environment was created by the researcher to gain the participants’ trust. Peer review was done and research findings were presented at a colloquium.

## Ethical considerations

Before the data were collected, the study was approved by the Postgraduate Research Ethics Committee and the Faculty Research Committee of the Tshwane University of Technology (FCRE-SCI) (clearance number: FCRE 2017/10/07 [SCI]). The Mpumalanga Research Committee and the chief executive officer of the Emerlo hospital also granted approval.

The participants were provided with full information on what the study entailed and what was expected from them. Informed consent was obtained from each participant. According to Saunders, Kitzinger and Kitzinger (2015:617), anonymity means that participants will not be traceable from the data presented about them. In this study anonymity was ensured by not using participants’ name. Instead, pseudonyms were used. The interviews were conducted in a private room with only the researcher and participant present. The information provided by the participants was not shared or discussed with any other person other than the supervisor and research committee.

## Findings

Five themes emerged from the study, namely, being hurt emotionally, acceptance, sense of guilt, support for mothers and community reaction to the congenital abnormality (see Table 2).

**TABLE 2:** Emerged themes.

Theme	Categories	Sub-categories
Being hurt emotionally	Disappointment	Unhealthy child Young mother dropping out of school Lack of information on diagnosis
	Pain	Comorbid condition as a young mother Sense of loss
Acceptance	Denial Disbelief	Conflict with paternal family Difficulty accepting the child
Sense of guilt	Self-blame Dissatisfaction Frustration	Should have terminated the pregnancy Questioning
Support for mothers	Absence of Psychological support	Support groups
Community reaction to the congenital disability	Poor social well-being	Child not growing Different from other children Not reaching milestones Struggling to get a crèche

### Being hurt emotionally

Participants expressed that they experienced ongoing disbelief and emotional pain from delivering children with congenital abnormalities. The study revealed that mothers were hurt emotionally by the birth of children with congenital abnormalities. In some cases, this was exaggerated by the delayed diagnosis and lack of information about the abnormality. Furthermore, some mothers were hurt emotionally by the fact that these children were their firstborns. Some of the mothers were also hurt emotionally by how their families reacted and responded to their children’s congenital abnormalities. Mothers expressed disappointment for delivering an unhealthy child. Mothers felt unprepared for nurturing and raising a child with a congenital abnormality. In addition, mothers anticipated a well-child who would develop according to milestones.

Below are some of the narratives from participants:

‘Then his family came and asked if in my family did we ever had such a condition? I said no. That really hurt me a lot because it was like they were not accepting my child. That really hurt me; I did not even tell his father. It hurts because you do not expect an unhealthy child at birth.’ (Thabile, 26 years old, Grade 12)

‘It hurts because you do not expect an unhealthy child at birth.’ (Ntokozo, 36 years old, Grade 10)

‘It hurts so much because I thought I will have a longer period with my child. However they had told me in hospital that a child with Down syndrome might not live long.’ (Nomthandazo, 20 years old, Grade 12)

‘It hurts because at first I did not know what was the problem as time goes by he got sick and I took him to the hospital. I was then told he is disabled and will not be able to do anything.’ (Maria, 40 years old, Grade 8)

‘I did not have high blood pressure but I developed it because having a child with congenital abnormality really hurts me, now I am on hypertensive medication.’ (Zodwa, 30 years old, Grade 12)

‘I was disturbed in a way that even now it still hurts. This affected because I even had to drop out of school in grade 11.’ (Mbali, 21 years old, Grade 11)

### Sense of guilt

Guilt was the second theme that emerged from the study findings. The mothers felt it was their fault that their children were born with congenital abnormalities. Moreover, they had unanswered questions about their children’s congenital abnormalities. The mothers also felt that their experience of motherhood was shortened because of delivering a child with congenital abnormality, especially if it was their first child.

Below are the narratives from some participants:

‘Sometimes I would blame God, asking why I get such a child as my first child.’ (Thembi, 27 years old, Grade 12)

‘I had so many questions why me? In my youth and it is my firstborn.’ (Anele, 20 years old, Grade 11)

‘I said amongst everybody else why me? What have I done wrong?’ (Maria, 40 years old, Grade 8)

‘I asked myself a lot of questions why me? What is it? Is it a gift but I did not have the right answer?’ (Zodwa, 30 years old, Grade 12)

‘I asked myself why do I get such a child as my firstborn, but I told myself maybe I will he will be fine once he start attending physiotherapist but he never did.’ (Ntokozo, 36 years old, Grade 10)

‘I felt bad for having a child with congenital abnormality, like it was my fault. I thought maybe I should have aborted but the other side felt I did right by not terminating the pregnancy.’ (Nomthandazo, 20 years old, Grade 12)

### Acceptance

The third theme that emerged from the study findings was acceptance of the child with congenital abnormality by the mother and family. The study found that some families accepted the children and support the mothers in raising them. However, other families made it difficult for the mother to accept her abnormal child because of the remarks towards her. It has been shown that it takes time for mothers to accept their children when they are born with congenital abnormalities. One participant stated that her experience was not so bad because of the presence of people who were born with congenital abnormalities in the family.

The study also showed that acceptance is still a challenge for mothers who have given birth to children with congenital abnormalities. Moreover, this is influenced by many factors, including acceptance by their family, peers and community at large. The study shows that mothers who are adequately and sufficiently supported tend to accept the condition more easily than those without support.

Below are some narratives regarding this:

‘His family came, they asked if in my family we ever had such a condition, it was like they doubt my child’s identity. They even said I should hide the hand of my child when walking in public, what will people say?’ (Thabile, 26 years old, Grade 12)

‘It hurts because her father’s family did not like her. Her uncle even said a child with mutism is not from our family. We do not have such in our family. In addition to that my sister sometimes shouts at my child and beats her with a mop, she would even say my child is a nuisance.’ (Thembeka, 35 years old, Grade 12)

‘The family have accepted him; they look after him and feed him because he doesn’t eat like any other child.’ (Maria, 40 years old, Grade 8)

‘I felt bad, but because from his father’s side there are people born with congenital abnormalities it was not that much bad. His uncles also have children with congenital abnormalities. Having a child with congenital abnormality did not affect me that much because both in my family and father’s side they are people who were born like that. I had support from both families.’ (Thobeka, 22 years old, Grade 12)

### Support for mothers

The fourth theme that emerged from the study was support from family. It was observed that most of the mothers derived support from their biological families, while others were supported by their in-laws. However, even with support from their families, the mothers still found it difficult to cope with having children with congenital abnormalities.

Below are some narratives from participants:

‘I get support from my parents and my brothers. They look after my child if I am not in the house.’ (Anele, 20 years old, Grade 12)

‘When my neighbours said a lot of things about my child, I told my sister and the father of my child because I do not have parents. They said I should not worry about what people say because people will always talk.’ (Zodwa, 30 years old, Grade 12)

‘I cannot say much about my family. I do not have a mother. I did not know how to care for a child who doesn’t know how to do anything. I was getting support from his father’s family sometimes.’ (Ntokozo, 36 years old, Grade 10)

‘I was supported by my mother; I even told her when my daughter’s uncle said that she is not their child. She told me not to worry she will teach me how to care for a child with disability.’ (Thembeka, 35 years old, Grade 12)

### Community reaction to the congenital disability

The fifth theme that emerged from the study findings was community reaction to the congenital abnormality. The study revealed that community members reacted and responded differently to the mothers of children born with congenital abnormalities. Some neighbours were supportive and assisted the mothers in recognising that their children were not growing well. Other neighbours were concerned about knowing why these children were not playing, not walking and not talking like other children. In addition, community members would question the mother as to what happened to the child.

Below are some narratives from participants:

‘I was told by my neighbour that my child is not growing well. She told me to [take] him to the clinic. However, some of my neighbours say a lot of things, they ask why is my son always on my back?’ (Zodwa, 30 years old, Grade 12)

‘My neighbours say Thomy is always on my back. He doesn’t grow. If it was me I would have killed him, so you cannot take all that people say. You just have to focus on your child and look forward to him getting better.’ (Maria, 40 years old, Grade 8)

‘The family even said I should hide his hand in public what will people say? I asked why because people should be used to him like this.’

She further explained that when she was in the community people kept asking what happened to the hand:

‘I do not know how to answer because he was born like that.’ (Thabile, 26 years old, Grade 12)

‘They ask why my child is not playing, why is not doing this and that. I have to explain to them that my child is not like any other child.’ (Anele, 20 years old, Grade 11)

‘I would not know what the community members say about my child because I spend most of the time with her indoors. Therefore, even if they talk I would not know what they say.’ (Betty, 44 years old, Grade 11)

‘People are not the same; others will sympathise with you while others will be saying shame on your child. I do not like it when people pity my child because he has blood just like them. The only difference is that he has hydrocephalus. At first I did not understand the abnormality of my child because when in public they would say my child is bewitched.’ (Thembi, 27 years old, Grade 12)

‘I had a fight with a lady one day and she said to me your disabled child serves you right.’ (Mbali, 21 years old, Grade 11)

## Discussion

This study identified the need for support of mothers who have children with congenital abnormalities in the Gert Sibande district, Mpumalanga. The findings of the study indicated that mothers were negatively affected by the birth of children with congenital abnormalities. There were multiple factors that emotionally hurt mothers of children with congenital abnormalities. Mothers were hurt emotionally by having to raise and nurture an unhealthy child. Moreover, some mothers were affected by the congenital abnormality in that they had to drop out of school to care for the child. The study conducted by Dambi, Jelsma and Mlambo (2015:7) in Zimbabwe agreed with the findings as it states that caregivers of children with congenital abnormalities were likely to have lower educational levels and diminished opportunities to find employment. Some mothers were also hurt emotionally because of their first pregnancy not being uneventful. The current study revealed that mothers experienced denial, disbelief and had difficulty accepting their children because mothers anticipate giving birth to a healthy child. A study conducted by Singogo, Mweshi and Rhoda (2015:5) in Zambia confirms that in some cases, family members fail to accept children with congenital abnormalities.

Mothers who participated in this study experienced disbelief and self-blame. Furthermore, they indicated that having a child with congenital abnormality not only impacted their psychological well-being but also impacted their physiological and physical aspects. The current study revealed that mothers experienced guilt for having children with congenital abnormalities. Moreover, a study conducted by Abbasi et al. (2016:82) in Iran confirmed that mothers show high levels of stress, anxiety, negative emotions, self-blame and fear of child’s future problems. The current study revealed that mothers experienced absence of psychological support. In addition, mothers felt they would have coped better if they had received proper counselling from nurses at the time of diagnosis. Moreover, mothers reported that having support groups for mothers who have children with congenital abnormalities would be very beneficial. In addition, such support groups will assist mothers to understand their children’s conditions better and strengthen their coping skills. A study conducted by Choi and Riper (2016:295) in Korea confirmed that parents who receive information and resources at the time of diagnosis tend to have a positive emotional response to the condition.

### Limitations

This study was of a contextual nature. It was conducted in a regional hospital in the Gert Sibande district using a purposive sampling method. Therefore, the results of the study are only applicable to that area and cannot be generalised and applied to other contexts.

### Recommendations

The nursing curriculum should emphasise on rehabilitation and training of nurses to support mothers of children who have congenital abnormalities.

Nurses should provide family-friendly services to accommodate all family members and encourage involvement in caring for children with congenital abnormalities as well as continued support for mothers.

## Conclusion

Studies have been conducted on the experience of mothers who have given birth to children with congenital abnormalities in other provinces. However, there was no evidence that such studies had previously been conducted in Mpumalanga, Gert Sibande district. This study is distinctive because it was conducted in an area with no apparent research outputs and where no study of such a calibre had previously been conducted. The study indicated a need to strengthen mothers’ support post-delivery of mothers of children with congenital abnormalities. Major concerns noted in the study were support of mother’s acceptance of the child by the mother, family and community, social isolation and lack of proper disclosure by health care professionals. This study showed a definite need for support of mothers in the society. This study indicated that families should be involved in the disclosure of their child’s condition to enhance care and support for mothers of children with congenital abnormalities. Mothers of children with congenital abnormalities are a vulnerable population. Therefore, they require intensive and adequate support, which is inclusive of physical, psychological and emotional support.
